# Rare enough ? Cardiac Device‐related pocket Infection due to *Mycobacterium fortuitum*

**DOI:** 10.1002/ccr3.3829

**Published:** 2021-01-26

**Authors:** Reshma Golamari, Nitasa Sahu, Rama Vunnam, Dhirisha Bhatt, Rameet Thapa, Ravi Patel, Rohit Jain

**Affiliations:** ^1^ Assistant Professor of Medicine Department of Hospital Medicine Penn State Health Milton S. Hershey Medical Center Hershey Pennsylvania USA; ^2^ Department of Infectious Disease UPMC Western Maryland Cumberland Maryland USA; ^3^ Resident Physician Department of Internal Medicine Penn State Health Milton S. Hershey Medical Center Hershey Pennsylvania USA

**Keywords:** Cardiac device infections, *M fortuitum* device infections, pocket infections, rapidly growing mycobacterial infections, rare device infections

## Abstract

Nontuberculous mycobacteria are rare causes of cardiac implantable electronic device (CIED)‐related infections and may lead to device‐related endocarditis, so preventing them is key. We present a case of CIED‐related pocket infection due to Mycobacterium fortuitum which highlights the challenges in management of such infections.

## Graphical abstract

It is important to consider nontuberculous mycobacteria as causes of cardiac implantable electronic device‐related infections, their treatment is challenging. These infections require prolonged therapy with antibiotics in addition to device removal.

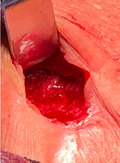

**Abbreviations**
CIED: cardiac implantable electronic device
CRT: cardiac resynchronization devices
NTM: nontuberculous mycobacteria
OR: operating room
RGM: rapidly growing mycobacterium

## 1 INTRODUCTION

Most cardiac implantable electronic device (CIED)‐related infections are pocket infections, and the most common organisms implicated in these are coagulase‐negative staphylococcus followed by *Staphylococcus aureus*.^1^ Infections caused by nontuberculous mycobacteria (NTM) are rare accounting to 0.2% of all CIED‐related infections, even though the bacteria are found ubiquitously in nature including soil, dust, and water.^2^, ^3^ Nontuberculous mycobacteria are divided into 4 groups—photochromogens, scotochromogens, nonchromogens (these are slow‐growing bacteria which take more than 2 weeks to grow in culture medium), and rapidly growing mycobacterium (RGM) which takes 3‐5 days to grow.^4^ *Mycobacterium fortuitum* is one the most commonly seen RGM species among > 100 species which have been identified and it spreads by natural or processed water, may produce infections if the medical equipment is cleaned with unsterilized water.^2^ They are capable of a whole spectrum of clinical diseases ranging from the more common prosthetic joint/surgical site infections to the rarely described CIED infections.^5^ NTM infections are emerging in unrecognized settings and are manifesting with new clinical signs and symptoms.^6^ The most common etiology of infection is the contamination of leads or the pulse generator during implantation or subsequent manipulation.^7^ Early‐onset CIED infections are from direct inoculation of the organism into the pocket whereas late onset usually results from reactivation disease, mycobacteremia, secondary seeding of the device.^5^ Important risk factors may be divided into patient‐related factors, such as end‐stage renal disease, procedure‐related risk factors such as the presence of hematoma among others and device‐related factors of which an abdominal pocket is an important one when compared to the pectoral since there are differences between the number of incisions, local vascularity, and local flora.^8^ These risks factors help stratify into high vs low risk of procuring the infection. We present one such case of early‐onset mycobacterial infection a month after a device placement in a low‐risk patient.

## 2 CASE

70‐year‐old male patient with recent history of a non‐ST elevation myocardial infarction with cardiac arrest status post two vessel coronary artery bypass grafting with course complicated by sustained ventricular tachycardia leading to CIED placement (dual‐chamber implantable cardioverter‐defibrillator with pacemaker function) presented to the hospital with complaints of redness at the site of his insertion pocket one month after implantation. There was also some purulent drainage from the insertion site few days prior to presentation. He did not have any fevers or chills. Upon presentation to the emergency department, the vital signs revealed a heart rate of 79 beats per minute, respiratory rate of 20 per minute, blood pressure of 125/79 mmHg, oxygen saturation of 97% on room air, and temperature of 97.5°F. Physical examination was significant for erythema at the CIED insertion site with tenderness to palpation and expression of purulent fluid. Cultures were sent from the emergency department. Laboratory data showed normal white cell count of 5100 per microliter and mild thrombocytopenia with platelet count of 121,000 per microliter. The remainder of the laboratory results was normal. The human immunodeficiency virus test was negative.

Chest X‐ray showed intact CIED leads without any acute abnormality. He was empirically started on intravenous vancomycin and cefazolin. Transthoracic echocardiogram was negative for vegetations on the native valves. Cardiology was consulted, and the device including the leads was subsequently removed with wound cultures obtained. During the procedure, copious amount of purulent drainage was noted. Gram stain showed moderate white blood cells without any organisms. The remainder of his clinical course was uncomplicated without any systemic signs of infection and blood cultures remaining negative. He was thus discharged with a life vest and was prescribed Doxycycline with outpatient infectious disease follow‐up. During follow‐up, the cultures were growing *M fortuitum* and he was thereafter started on Levofloxacin and Clarithromycin. This was eventually changed to Levofloxacin and Bactrim after susceptibility results were available (Table 1). He was treated for 4 months, and the infection resolved.

**Table 1:** Antimicrobial susceptibility results on 2/5/2019

Antibiotic	MIC(mcg/mL)	Interpretation
Amikacin	<1	Susceptible
Cefoxitin	64	Intermediate
Ciprofloxacin	<0.12	Susceptible
Clarithromycin	16	Resistant
Doxycycline	>16	Resistant
Imipenem	8	Intermediate
Linezolid	4	Susceptible
Moxifloxacin	<0.25	Susceptible
Tigecycline	0.25	Susceptible
Tobramycin	16	Resistant
Trimeth/Sulfa	1/19	Susceptible

**Figure 1:**
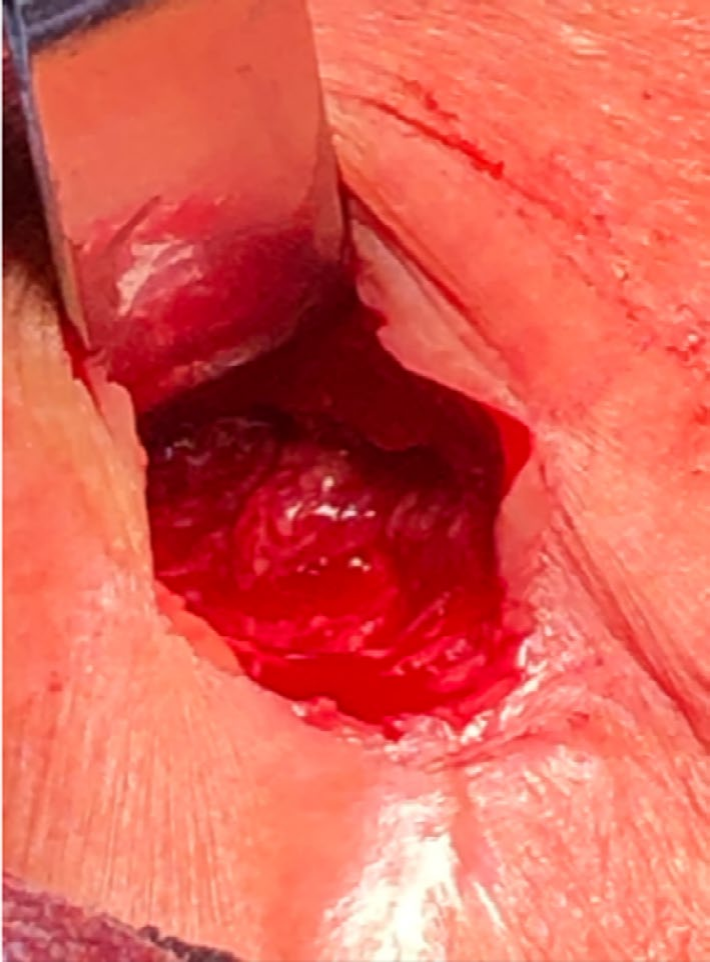
Incision and drainage of the pocket infection

However, at one of his follow‐up visits 5 months later, he was noted to have tenderness, redness, and swelling of his left chest wall again. An ultrasound was ordered which showed a possible pocket of abscess collection, approximately 4.7 x 3.0 cm in size, and he was subsequently admitted to the hospital. He was empirically started on Vancomycin and Ceftriaxone, and repeat blood cultures were sent. The patient was then taken to the operating room for incision and drainage (Figure 1), and wound cultures were obtained. Considering his recurrence and based on prior antibiotic susceptibilities, the patient was discharged on Bactrim and Linezolid. However, he developed pancytopenia that was attributed to Linezolid, so he was switched to a combination of Ciprofloxacin and Bactrim. His wound cultures grew *M fortuitum* this time as well, and he was able to complete his antibiotics without further complications. He was eventually seen in the clinic, for a follow‐up at a six‐month and deemed to be infection‐free based on clinical symptoms, and eventually was able to get a CIED placement on his right side.

## 3 DISCUSSION

The incidence of CIED infections has increased almost 96% between 1993 and 2008, and more recent data until 2015 as studied by Dai et al. confirmed an increased trend within the last two decades.^9^, ^10^ The incidence of pocket infections without blood stream infections was noted to be 1.37 per 1000 devices and defibrillators were associated with an increased risk when compared to pacemakers.^11^ This may be related to larger sizes of the cardiac resynchronization devices and longer duration of surgery which may contribute to infection. In the modern era, cosmetic procedures, tattooing, and pedicures account for more mycobacterial infections when compared to surgical site procedures.

The most common predisposing factor for device‐related endocarditis is the presence of pocket infection^1^ and that is why controlling the rate of pocket infection becomes crucial. Pocket infections can also complicate and cause cutaneous erosion of the generators, and the bacteria may translocate from the pocket infection through the electrodes. The risk factors for these infections include immunocompromised status, diabetes mellitus, and chronic renal insufficiency.^12^

Gram‐positive bacteria are the predominant causative pathogens, both early‐ (<1 year from device implantation) and late‐onset infection. Coagulase‐negative staphylococci and *Staphylococcus aureus* remain the most common pathogens, which account for 70% of the infections.^13^ Gram‐negative pathogens account for 6%‐9% of infections.^3^ Rapidly growing mycobacteria are common environmental organisms which have been isolated from soil, food, natural water, various plants/animal surfaces, and hospital surfaces.^2^ The species of RGM capable of affecting humans consist primarily of the *Mycobacterium fortuitum* group, the *M chelonae/abscessus* group, and the *M smegmatis* group.^2^ They are known for presence of fatty acids known as mycolic acids and they can produce visible colonies within 7 days of growth on solid medium.^4^ Among the various species, most commonly encountered RGM species are *M fortuitum* and *M abscessus/M chelonae*.^12^

Our case highlights the challenges in treating a pocket infection that can be caused by a RGM such as *M fortuitum*. Majority of the infections occur < 6 months after implantation, and our patient was one of them. *M* *fortuitum* usually takes >= 5 days to appear on the standard medium, exceeding the incubation time of routine cultures leading to a missed opportunity for a diagnosis. Even when growth is observed, these organisms appear as gram‐positive bacilli on gram stain, and can be misinterpreted as a contaminant or *Corynebacterium* or *Nocardia* species.^5^, ^14^

Although these bacteria grow within 3‐7 days on standard agar blood culture media, cultures may not yield these mycobacteria since they may not be held long enough to demonstrate the growth. Swab samples from wounds maybe difficult to culture; some authors have suggested the use of skin punch biopsies to capture these organisms. Presence of acid‐fast bacilli on smears may aid in the diagnosis. Accurate diagnosis requires PCR‐restriction enzyme analysis (PCR‐REA).^15^ The absence of constitutional symptoms such as fever, chills, and rigors further complicates the difficulty in diagnosing these infections.

Although recent evidence suggests the use of an antibacterial envelope containing rifampin and minocycline to mitigate major infections, this may not be effective against NTM infections. These infections do not respond to the common antituberculous drugs as well. These require a prolonged course of treatment and even wound debridement to eliminate these infections.^15^ Identification of the subspecies is important since they have predictable antimicrobial susceptibility patterns.^16^ M *fortuitum* is generally more drug susceptible when compared to the other species and often oral regimens can be devised. Therapy with tetracyclines, sulfamethoxazole, and quinolones is usually effective.^16^ However, RGM can develop macrolide resistance by mutations in the peptidyl‐transferase region of the 23S ribosome gene.^17^ Some experts opine that macrolide should not be used in empiric therapy. However, good results have been obtained when macrolides are combined with one other additional drug if they demonstrate in vitro susceptibility.^16^ A variety of drug combinations have been used that includes Ofloxacin, Clarithromycin, Amikacin, Sulfonamides, Ciprofloxacin, Doxycycline, and Linezolid to deal with these infections.^15^, ^17^ The duration of treatment ranges from 3 months to longer durations. Pulmonary infections require a more prolonged treatment course, sometimes upto a year. More often than not, with these infections, it is strongly recommended that entire device or hardware be removed urgently. An incomplete removal of the device may result in higher relapse rates. The American Heart Association statement recommend complete removal of all hardware (Class 1 evidence) from patients, even in the absence of systemic infections signs.^12^ Heart Rhythm Society also recommends complete course of antibiotics especially after complete device and lead removal in patients with pocket infections (Class 1 indication).^18^ Usually the recommendation is for implantation of the CIED on the contralateral side. The optimal interval between device replacement after removal is not defined and is dependent on the extent of infection. Attempts to reuse the pacemaker/defibrillators have resulted in infections even on the contralateral side.^19^ Moreover, the usual methods of operating room sterilization are not sufficient for eradicating mycobacteria.

For soft tissue infections due to *M fortuitum*, a minimum of 4 months of therapy with at least two agents with in vitro activity against the clinical isolate is necessary to provide a high likelihood of cure. A longer duration of treatment generally is required because of the propensity of the organism to cause a biofilm. Our patient was treated with levofloxacin and Bactrim for 4 months. It is important to monitor for adverse reactions of antibiotics since they are required for longer duration.

In conclusion, CIED pocket infections caused by *M fortuitum* infections are rare, often misinterpreted at the time of diagnosis. Once diagnosed, combination therapy is better to avoid treatment failure due to development of drug resistance when compared to monotherapy. Removal of the entire device is recommended.

## 4 ETHICS STATEMENT

All the authors collectively agree that the manuscript is original and has not been published elsewhere.

## DISCLOSURE STATEMENT

The authors do not have financial relationships with any commercial entity that has an interest in the subject of the presented manuscript or other conflicts of interest to disclose.

## AUTHOR CONTRIBUTION

Reshma Golamari: involved in design of the manuscript, revisions and finalized the draft. Nitasa Sahu and Rama Vunnam: contributed to the manuscript draft. Dhirisha Bhatt and Ravi Patel: contributed to manuscript revisions. Rameet Thapa: involved in data acquisition, finalized the draft, contributed to manuscript revisions, and provided feedback. Rohit Jain: involved in data acquisition and finalized the draft.

## Data Availability

All data analyzed in this study are included in the article.
